# Developing a National Set of Health Equity Indicators Using a Consensus Building Process

**DOI:** 10.34172/ijhpm.2021.54

**Published:** 2021-06-23

**Authors:** Rachel Wilf-Miron, Shlomit Avni, Liora Valinsky, Vicki Myers, Arnona Ziv, Gidi Peretz, Osnat Luxenburg, Mor Saban, Paula Feder-Bubis

**Affiliations:** ^1^The Gertner Institute for Epidemiology and Health Policy Research, Sheba Medical Center, Ramat Gan, Israel.; ^2^School of Public Health, Sackler Faculty of Medicine, Tel Aviv University, Tel Aviv, Israel.; ^3^Strategic and Economic Planning Administration, Ministry of Health, Jerusalem, Israel.; ^4^Public Health Services, Ministry of Health, Jerusalem, Israel.; ^5^Medical Technology, Health Information and Research Directorate, Ministry of Health, Jerusalem, Israel.; ^6^Department of Health Policy and Management, Faculty of Health Sciences & Guildford Glazer Faculty of Business and management, Ben Gurion University of the Negev, Beersheba, Israel.

**Keywords:** Consensus Building, Delphi Technique, Health Equity, National Equity Indicators, Health Disparities

## Abstract

**Background:** Considerable health inequities documented in Israel between communities, populations and regions, undermine the rights of all citizens to optimal health. The first step towards health equity is agreement on a set of national indicators, reflecting equity in healthcare provision and health outcomes, and allowing monitoring of the impact of interventions on the reduction of disparities. We describe the process of reaching a consensus on a defined set of national equity indicators.

**Methods:** The study was conducted between January 2019 and June 2020, in a multistage design: (A) Identifying appropriate and available inequity measures via interviews with stakeholders. (B) Agreement on the screening criteria (public health importance; gap characteristics; potential for change; public interest) and relative weighting. (C) Constructing the consultation framework as an online, 3-round Delphi technique, with a range of experts recruited from the health, welfare and education sectors.

**Results:** Participants were of diverse age, gender, geographic location, religion and ethnicity, and came from academia, healthcare provision, government ministries and patient representative groups. Thirty measures of inequity, presented to participants, represented the following domains: Health promotion (11 indicators), acute and chronic morbidity (11), life expectancy and mortality (2), health infrastructures and affordability of care (4), education and employment (2). Of the 77 individuals contacted, 75 (97%) expressed willingness to participate, and 55 (73%) completed all three scoring rounds. The leading ten indicators were: Diabetes care, childhood obesity, adult obesity, distribution of healthcare personnel, fatal childhood injuries, cigarette smoking, infant mortality, ability to afford care, access to psychotherapy and distribution of hospital beds. Agreement among raters, measured as intra-class correlation coefficient (ICC), was 0.75.

**Conclusion:** A diverse range of consultants reached a consensus on the most important national equity indicators, including both clinical and system indicators. Results should be used to guide governmental decision-making and inter-sectoral strategies, furthering the pursuit of a more equitable healthcare system.

## Background

**Key Messages**

**Implications for policy makers**

- Efforts to reduce health inequities between population groups require the collaboration of multiple stakeholders who must prioritize the most important domains for intervention.
- Consensus was reached on ten selected national indicators, representing preventive and chronic healthcare, and health system factors.
- Both experts and public representatives from a wide range of sectors should be consulted when rating evidence-based equity indicators.
- Active participation of influential stakeholders in the consensus process increases the chances of implementation of the indicators selected in order to reduce gaps and increase health equity.

**Implications for the public**

Health equity implies that everyone should have a fair opportunity to attain their full health potential, regardless of gender, ethnicity, geography, and social or economic status. Health inequities exist, with disadvantaged groups suffering poorer health outcomes. In order to reduce these inequities, countries must prioritize which topics are the most urgent to address, with limited resources. We designed and implemented a consultation process involving experts and public representatives from a broad range of sectors who were asked to rate the relative importance of 30 health equity indicators and to reach a consensus on the most important ones. The ten indicators selected included childhood and adult obesity, cigarette smoking, child accidents, infant mortality, diabetes care, distribution of hospital beds and health personnel, and access to psychotherapy. Resources should be invested in these areas in order to contribute to a more equitable healthcare system.

Equity in the provision of care, regardless of gender, ethnicity, geography, and social or economic status, was defined by the Committee on Quality of Health Care in America as one of the six dimensions of quality of care.^1^ Health equity implies that everyone should have a fair opportunity to attain their full health potential.^2^ Since the publication of the Commission on Social Determinants of Health (SDH) report in 2008,^3^ the equity discourse has related to this theoretical framework that explains the underlying processes that underpin health inequities. The framework includes three core components: (1) the socioeconomic and political context; (2) structural determinants of health inequities, for example social class divisions; and (3) intermediary determinants of health – housing, physical work environment, nutrition and physical activity. The structural determinants operate through intermediary determinants of health to shape health outcomes. As an “action oriented” framework, it is designed to help policy-makers pinpoint where to intervene to most effectively tackle health inequities.^4^ Inequity in access to healthcare, education, decent work and living conditions, developed neighbourhoods, healthy communities, trust and a sense of belonging, are major contributors to health inequities.^3,5,6^ Therefore, efforts to reduce health inequities must also take into account the effects of education, income, ethnicity and geography on health outcomes. Monitoring health equity is guided by values, including the basic human right to health and non-discrimination.^5^ Reducing health inequities is a moral duty of governments and healthcare organizations. Governments have set up national health-equity surveillance systems for routine monitoring of health inequity and the SDH, and to evaluate the impact of health equity policy and interventions. Many countries, like Canada, New Zealand, the United States and United Kingdom have implemented measurement systems, based on an agreed set of indicators, allowing monitoring of the impact of interventions on the reduction of inequities between disadvantaged groups and the rest of the population.^7-10^

 Israel is characterized by considerable income inequalities, as manifested by a high Gini coefficient, compared to the majority of Organisation for Economic Co-operation and Development (OECD) countries.^11^ National expenditure on health and its percentage of the gross domestic product is considerably lower than the OECD average. Health expenditure from public sources as a share of total spending, is lower compared with the OECD average (64% and 71% respectively),^12^ resulting in a growing burden of out-of-pocket expenditure on health services. This affects socio-economically disadvantaged households the most,^13^ especially those in peripheral areas. Israel is a multi-ethnic country, with Jews comprising 74%, Arabs, mostly Muslim – 21%, and others – 5% of the total Israeli population.^14^ Despite its small size, two regions (North and South) are regarded as the periphery of the country. Both comprise small urban and rural communities, lower access to services, with lower socioeconomic status (SES), higher unemployment rates and higher proportion of populations at-risk for inequity, such as immigrants and Arabs, compared with the central urban regions.^15^

 Israel has a public healthcare system, framed by the National Health Insurance Law that mandates a defined, comprehensive basket of health services to all citizens. Services are provided by four not-for-profit health maintenance organizations (HMOs).^13^ Since 2004, a National Program for Quality Indicators in Community Healthcare provides policy-makers and citizens with information on the quality of community healthcare in Israel. This yearly report, based on data from more than 90% of all Israeli citizens, includes several dozen indicators in health promotion and care for non-communicable diseases.^16^

 In Israel, academic and national bodies, such as the Central Bureau of Statistics, have documented inequities related to education, income and ethnicity.^9^ The Ministry of Health (MoH) publishes a yearly report on health inequities, since 2010.^17^ Considerable disparities have been consistently reported, for example in infant mortality, risk factors like smoking and obesity, non-communicable diseases and life expectancy, between the Arab and Jewish populations,^18,19^ and income-related disparities in health services utilization and health outcomes.^20^ Other population groups identified as at-risk for health inequities are ultra-Orthodox Jews, and immigrants from countries of the former Soviet Union and from Ethiopia.^13^ These yearly reports include analysis of regional inequities, highlighting the gaps between the center and the periphery.^15,21^ Despite diverse efforts,^22–25^ mainly by the MoH and the four HMOs, considerable health gaps still exist between communities, populations, and regions of the country.^26^ These gaps, coupled with a high national level of poverty and widening social disparities,^27^ led the MoH to define equity measurements as part of its strategic work plan. In 2018, the MoH decided on the development of a set of national equity indicators, to guide its strategy towards more equitable health outcomes and allow regular monitoring of progress in the reduction of inequity in both health outcomes and access to health services.

 Social participation in decision-making is a key driver for the promotion of health equity.^28^ In light of this understanding, a Delphi technique was utilized to shape a collaborative, multidisciplinary, and multi-sectoral process to choose the set of national health equity indicators.^29^ Delphi is a widely used and accepted technique for systematically gathering and combining data from a group of respondents within their area of expertise, in order to arrive at an informed consensus on a complex problem.^30^ The technique is designed to achieve a convergence of opinion on a specific real-world issue, using iterative rounds of grading with controlled feedback reports from the research team. Participants have the option to reconsider their initial grading in light of the group scoring. This technique has been widely used to reach agreement among experts and/or public representatives on various issues, such as health policy,^31^ patient reported outcome measures,^32^ as well as in selection of equity^33^ and quality^34^ indicators.

 The objective of this manuscript was to describe the process, of reaching a consensus among experts and public representatives on a defined set of evidence-based national indicators, aimed to monitor equity, or lack thereof, in health services provision and health outcomes.

## Methods

###  Study Design and Setting

 The study was commissioned by the MoH, which recently defined the development of quantifiable equity measures as part of its strategic work plan. The research team included the MoH’s senior representative (SA), a social worker andequity promotion expert; experts in quality management (RWM, LV, physician and nurse), equity promotion (RWM) and health promotion (LV), quality measurement and methodology (PFB, a sociologist).

 The Israeli National Institute for Health Policy Research provided both the finance and a steering committee to guide the research. This committee was composed of 10 senior members from the fields of health equity (4 members, one of them a professor of social work), economics (1), health policy (2) and quality measurement (3).

 The steering committee met with the researchers in person (frontal meetings) for the purpose of designing and monitoring the process as well as for summarizing the outputs of the process and the lessons learnt, once the consultation ended.

 The multi-stage study took place during January 2019-June 2020 and consisted of the following stages:

###  Stage A – Identification of Potential Equity Measures

 A1. *Exploring diverse data sources:* Interviews were conducted with stakeholders and data holders such as the directors of national registries at the Israel Centre for Disease Control, to map all available sources of indicators. The guiding principles in choosing the stakeholders were the relevance of data managed in their organization to the health of the population, their ability to provide data, and sector diversity, so as to include education and welfare stakeholders, in addition to those from the healthcare sector. All interviews comprised a semi-formal dialogue including (1) presentation of the aim of the research and what kind of data is needed, (2) learning about data availability in the domain or organization under consideration, and (3) defining what kind of data is required. The interviews were conducted by one of the researchers (RWM), considering that an open, sincere dialogue was the best way to achieve cooperation from the stakeholders.

 A2. *Defining the characteristics of a “good” indicator for the measurement of equity*: In view of the interview findings, and based on the relevant literature, potential indicators were searched for, based on the following characteristics: Indicators of good quality,^35,36^ according to their significance to the Israeli healthcare system^37^ (ie, that measures an issue of public health importance, based on the clinical meaningfulness of the domain under consideration, and the size of the population affected); and solid scientific basis (ie, widely-accepted evidence base); reliability (ie, data is less amenable to manipulation); feasibility of data retrieval (preferably computerized data that is routinely collected, rather than data that requires active collection). The researchers and the steering committee based their definition of a “good indicator” on their 15-year-experience gained in national quality measurement,^37^ a program that two of the researchers (LV and RWM) actively participated in, and the head of the steering committee led for a decade. Historical data of at least three consecutive years was regarded as an optional requirement for a “good” equity indicator, allowing evaluation of whether the gap widened, narrowed or remained stable over time. In fact, none of the indicators for which there were time trends data, demonstrated narrowing of the gap over time.

 A3. *Defining the SDH to guide data analysis*. The following determinants were chosen, based on international experience^7-9,38^ and data availability: age, sex, ethnicity, religiosity, immigration, and SES. Data on each indicator were presented to participants as differences between population groups, including male vs female, Arabs vs Jews, older vs younger, secular vs ultra-Orthodox, low to high SES quartile, and geographical centre vs periphery. Whenever data on ethnicity, immigration, income and education were unavailable at the individual level, proxy variables were calculated from the socio-demographic characteristics of the residential neighbourhood (N~3000 inhabitants), according to home address. An example is the proxy estimation of sociodemographic characteristics for all patients on dialysis for end-stage kidney disease during 2014-2018. The encrypted ID number of each individual patient was matched with the Internal Affairs Ministry’s registry of addresses. Each home address was translated using geo-coding (turning descriptive locational data such as a postal address or a named location into an absolute geographic reference)^39^ to a specific geographic statistical area, with its unique sociodemographic attributes. Ethnicity, religiosity and SES of the area were allocated to the individual patient.

 Only indicators with a relative difference of at least 10% between the at-risk population and others were included. These cut-off points were guided by the experience acquired in the two largest Israeli HMOs, Clalit Healthcare Services^40^ and Maccabi Healthcare Services.^41^

 The output of this first stage was a list of 30 equity indicators for presentation to participants in the consensus process. This number of measures was compatible with the literature about on-line Delphi techniques.^32,42^

###  Stage B: Agreement on the Criteria for Equity Indicator Rating 

 Based on the literature and on the experience gained in the extant Israeli National Program for Quality Indicators in Community Healthcare,^37,43^ the researchers identified and evaluated diverse criteria that could guide the Delphi participants on the rating of the inequity measures. The steering committee helped define the final four criteria. The relative weight of each criterion was based on anonymous voting by the steering committee members (n = 10). Scoring was on a scale of 0-100 points and the final weight for each criterion was the average of 10 scores. The selected criteria were: *public health importance* - the extent to which inequity in the specific health domain affects public health, and the size of population affected (relative weight 0.37); *gap characteristics*– the magnitude of the gap, the number of social determinants that shape the gap, its trend over time (0.26); *potential for change*– the ability of actions taken by the healthcare, education or welfare systems, to significantly reduce this gap (0.27); *public interest* - the importance of the issue and unfairness of the gap, as perceived by the public (0.10).

###  Stage C: Designing the Consultation Framework

 Participants – Three of the researchers (RWM, LV and SA) suggested a list of potential participants, based on their previous experience with experts from diverse and wide-ranging areas, each holding the relevant knowledge and information in their field of work or advocacy.^44^ After discussing the list of candidates among the researchers and consulting the steering committee, a list of 77 potential participants was proposed, aiming to include as diverse a range as possible, in terms of multi-sectorial – persons affiliated with the health, welfare and education sectors; multi-level – including senior and middle management employees, as well as direct service providers from the field; and diversity in terms of age, gender, geographic location and ethnicity. Among the 75 participants were experts in health economics, a leader of health policy and innovation in one of Israel’s HMOs, a leading professor of social work, the head of policy planning at the MoH, all 4 representatives of equity promotion in the 4 Israeli HMOs, patient and public representatives from the Israeli Mental Health Association and the Society of Patients’ Rights in Israel. These representatives were chosen due to their involvement in equity issues and ability to use digital platforms. Participants were not offered any incentive for participation.

 Of the 75 individuals who were willing to participate, 25 were from the academia (research institutes, universities); 23 were involved in healthcare provision (medium level management and healthcare professionals, both from community and hospital settings); 17 were affiliated with non-governmental organizations, both patient and public representatives; and 10 were affiliated with government ministries (Table 1).

**Table 1:** Participants’ Characteristics (n = 75)

	**No.**	**% **
Gender		
Female	49	65.3
Ethnicity		
Arab	7	9.3
Residence		
Geographic periphery	10	13.3
Major domain^a^		
Academia	25	33.3
Ministries	10	14.7
Patient representatives	17	22.7
Healthcare organizations	23	30.7

^a^Major domain – main place of work or reason for being invited to participate, ie, a medical director who is also a lecturer at a university is classified as “healthcare organization.”

 Special efforts were made to choose participants from the geographic periphery and the Arab ethnic minority. Despite these efforts, the geographic periphery and the Arab minority were under-represented (13.3% participants from the periphery vs 31% in the population; and 9.3% Arab participants vs 21% of the population). Participation was anonymous. Each participant was recruited by a personal phone call from one of the researchers.

 A three-round Delphi was chosen to reach a consensus over a set of indicators. To allow busy participants from the whole country to participate, an online process was undertaken, facilitated by *Qualtrics survey software*.^45^ The digital process allowed participants to rate the indicators individually, in their own time. Each inequity indicator was presented in a similar way: title and definition; description of relative differences by SDH - gender, age, SES, ethnicity, residence in the geographic periphery, and immigration – depending on data availability (Table S1, Supplementary file 1). When presenting the indicators, we used language that could be understood by diverse audiences, including laypersons and non-health professionals. Additional tables and diagrams were available to participants for more detailed, raw data (eg, the percent of smokers among Arab and Jewish males is 39% and 21.6% respectively). For each measure, participants were asked to rate all four criteria on a 5-point Likert scale, “1” indicated “not important at all” and “5” indicated “very important.” Rating of all indicators was mandatory, and participants could not continue to the next indicator until they had completed the scoring of the previous one, ensuring that all participants submitted full scoring sets.

 The Delphi took place between February and May 2020, with intervals of 2 weeks between the first and second rounds and 8 weeks between the second and the final (third) rounds. The latter was postponed due to the outbreak of coronavirus disease 2019 (COVID-19), which caused increased burden on healthcare staff and management and timetable constraints among most of the participants.

 In the first Delphi round, participants were invited to suggest additional indicators that they perceived to be important, to be included in the following 2 rounds. At the beginning of the second and third rounds, participants were informed of the scoring results from the previous round. One automated reminder was sent to non-respondents 5-7 days after the beginning of each round, followed by a personal reminder via SMS, by the researcher who recruited the participant. A graphic description of the process is presented in Figure. The middle column (Digital Delphi Survey) describes the number of indicators in each of the rounds.

**Figure:**
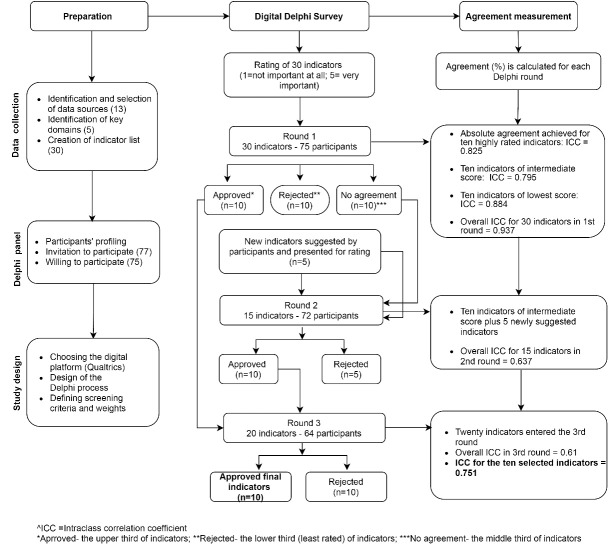
Flowchart of the Delphi Process for Selecting Equity Indicators.

###  Statistical Analysis

####  Calculation of Inequities Presented to Participants

 Data from diverse sources were translated and presented as relative difference measures, which express difference between a measure of interest (rate, percentage, mean or some other quantitative measure) and the chosen reference point.^46^ Relative difference was calculated as rate ratios, comprising the incidence rate in one group divided by the incidence rate in a comparison group. For example, in 2017, the prevalence of obesity among Arab females was 27.4%, while prevalence among Jewish females was 15.6%. We calculated the rate ratio as 27.4/15.6 = 1.76.

 Data were presented to participants as relative difference, since this is the most widely used measurement of inequities in Israel.^40,41^ Using this familiar measure in the consultation could not only further participation but also enhance the chances of implementation of the consensual indicators.

 Full data on the relative differences for each indicator are presented in Table S1. Differences are presented as rate ratios between ethnic groups, genders, regions, age groups, SES levels, and religious groups.

####  Agreement Between Participants

 Interrater reliability of the total score of each of the measures was quantified using an intra-class correlation coefficient (ICC) model (2,k), namely two-way random effects, absolute agreement and single rates/measurement. ICC demonstrates agreement between raters. This method was previously used in similar studies.^44^ Based on a 95% confidence interval of the ICC estimate, ICC values less than 0.5 are indicative of poor reliability, between 0.5 and 0.75 indicate moderate reliability, between 0.75 and 0.9 indicate good reliability, and values greater than 0.90 indicate excellent reliability.^47^

 Further analysis was conducted to estimate agreement between raters for each indicator, utilizing one-sample Kolmogorov-Smirnov normal test (Table S2).^48^

## Results

###  Creation of Inequity Indicators

 In-depth semi-formal interviews with data managers in the preparation phase revealed that data can be collected and pooled from multiple sources to create inequity indicators.

###  Indicators by Key Domain

 Thirty inequity indicators were presented to participants at the beginning of the process (presented in Table 2).

**Table 2:** Indicator Scoring, by Delphi Technique Round^a^

**Domain**	**Indicator Definition**	**Score in Round 1**	**Score in Round 2**	**Score in Round 3**
Health promotion and disease prevention	*Cigarette smoking *	4.14		3.79
	Child immunization	3.93	3.46	
	Dental hygiene, children	3.90	3.88	3.64
	Physical activity	3.79	3.6	3.51
	Screening for colon cancer	3.79	3.48	3.66
	Screening for breast cancer	3.71	3.57	3.66
	First visit at well baby clinic	3.61		
	Dental hygiene, adults	3.55		
	Loneliness	3.49		
	Antibiotic prophylaxis for hip fracture surgery	3.28		
	Perceived health status	3.13		
Acute and chronic morbidity	*Diabetes care*	4.24		4.09
	*Childhood obesity*	4.14		4.02
	Incidence and treatment of stroke	4.11		3.58
	*Adult obesity *	4.09		3.92
	Prevalence of end stage renal disease, requiring dialysis	3.80	3.75	3.47
	Lung cancer – stage of diagnosis	3.56		
	Prevalence and treatment of dementia	3.54		
	Post-natal depression	4.01		3.68
	Weight loss among the elderly	3.49		
	Breast cancer – stage at diagnosis	3.38		
	Severe physical disability	3.34		
	Colorectal cancer – stage of diagnosis	3.10		
Accessibility and affordability of healthcare	*Ability to afford care*	4.06		4.02
	*Distribution of healthcare personnel*	3.95		3.91
	*Access to psychotherapy *	3.90	3.72	3.77
	Distribution of hospital beds	3.83	3.82	3.74
	Burden of private financing for medical treatment	3.52		
Life expectancy and mortality	*Fatal childhood injury*	4.21		3.89
	*Infant mortality*	4.19		3.79
	Suicide	3.79	3.52	3.38
	Life expectancy	3.72	3.64	3.34
Education and Employment	Education	3.59		
	Employment	3.05		
Other	Incidence of violent offences		3.85	3.40

^a^The 10 Indicators with the highest scores in the third, final round, are marked in italics.

 These indicators represented the following 5 domains: health promotion and disease prevention (12 indicators), acute and chronic morbidity (10), mortality and life expectancy (2), healthcare resources, accessibility and affordability (4), and education and employment (2). For a full list of indicators and selected relative inequities, see Table S1.

###  Response and Delphi Participation Rates

 Of the 75 people who agreed to participate, 72 (96%) completed the first round. In the second and third rounds, 64 out of 72 (88.9%) and 55 out of 64 (85.9%), respectively, completed the survey. Thus, the overall Delphi response rate – 55 out of 75 people who agreed to participate (73.3%) completed the 3 rounds.

###  Indicator Rating

 The right column of Figure describes the agreement of the participants within each of the rating rounds.

Table 2 presents the overall weighted score of each of the 30 indicators, for each of the 3 Delphi rounds.

 The 10 leading indicators at the end of the third round, with the final score (in parentheses) were: diabetes care (4.09 on a 5-point-scale), childhood obesity (4.02), ability to afford care (4.02), adult obesity (3.92), distribution of healthcare personnel (3.91), fatal childhood injuries (3.89), cigarette smoking (3.79), infant mortality (3.79), access to psychotherapy (3.77) and distribution of hospital beds (3.74).

 Analysis of the scores allocated to each of the four screening criteria (Table 3), revealed that public health importance was given the highest score across all 30 indicators (average score 4.02) while potential for change received the lowest scores (average score 3.32). The character of the gap and public interest received intermediate scores (3.43 and 3.44, respectively).

**Table 3:** Indicator Scoring by Screening Criteria and Weighted Final Score

**Domain**	**Indicator**	**Weighted Final Score**	**Public Health Importance** ^1^		**Potential for Change** ^2^		**Gap Characteristics** ^3^		**Public Interest** ^4^	
			**Mean**	**SD**	**Mean**	**SD**	**Mean**	**SD**	**Mean**	**SD**
Health promotion and disease prevention	Cigarette smoking	4.14	4.63	0.61	3.58	0.85	4.06	0.79	4.07	0.88
	Child immunization	3.93	4.58	0.66	3.73	1.05	3.21	0.99	3.90	0.97
	Dental hygiene, children	3.90	4.14	0.79	3.93	0.94	3.76	0.86	3.32	0.93
	Physical activity	3.79	4.38	0.68	3.28	0.98	3.72	0.79	3.24	1.05
	Screening for colon cancer	3.79	4.37	0.76	3.77	0.83	3.17	1.01	3.32	1.00
	Screening for breast cancer	3.71	4.11	0.94	3.79	1.01	3.17	1.03	3.46	1.03
	First visit at well baby clinic	3.61	3.85	0.93	3.63	0.89	3.48	1.03	3.07	1.05
	Dental hygiene, adults	3.55	3.79	0.89	3.35	1.05	3.69	0.88	2.86	1.08
	Antibiotic prophylaxis for hip fracture surgery	3.28	3.67	1.01	3.59	1.15	2.66	1.08	2.60	1.09
	Perceived health status	3.13	3.42	1.03	2.55	0.96	3.41	1.07	2.92	1.04
Acute and chronic morbidity	Diabetes care	4.24	4.72	0.51	3.93	0.91	4.04	0.90	3.80	0.88
	Childhood obesity	4.14	4.54	0.65	3.89	0.91	3.92	0.70	3.96	0.86
	Incidence and treatment of stroke	4.11	4.59	0.70	3.59	0.91	4.04	0.91	3.92	0.87
	Adult obesity	4.09	4.62	0.61	3.52	1.06	3.97	0.84	4.01	0.85
	Post-natal depression	4.01	4.27	0.79	3.73	0.96	4.10	0.87	3.54	0.99
	Prevalence of end stage renal disease, requiring dialysis	3.80	4.31	0.70	3.28	0.97	3.80	0.85	3.27	0.86
	Lung cancer -stage of diagnosis	3.56	4.15	0.83	3.07	1.00	3.31	0.85	3.31	0.83
	Breast cancer - stage at diagnosis	3.38	3.96	0.97	3.23	1.18	2.69	0.97	3.39	0.91
	Severe physical disability	3.34	3.86	0.97	2.61	1.01	3.51	0.90	2.99	0.97
	Colorectal cancer - stage of diagnosis	3.10	3.77	1.10	2.83	1.13	2.49	0.98	2.96	0.89
Accessibility and affordability of healthcare	Ability to afford care	4.06	4.41	0.74	3.75	0.96	3.90	0.89	4.00	0.99
	Distribution of healthcare personnel	3.95	4.18	0.89	3.70	1.17	3.80	0.94	4.17	0.90
	Access to psychotherapy	3.90	4.21	0.87	3.79	1.09	3.66	1.07	3.69	1.06
	Distribution of hospital beds	3.83	4.11	0.94	3.51	1.21	3.65	0.89	4.21	0.92
Life expectancy and mortality	Fatal childhood injury	4.21	4.59	0.62	3.86	0.98	4.04	0.79	4.15	0.80
	Infant mortality	4.19	4.63	0.63	3.61	1.01	4.18	0.84	4.20	0.85
	Suicide	3.79	4.15	0.85	3.30	0.98	3.76	0.93	3.83	0.98
	Life expectancy	3.72	4.28	0.81	2.89	0.97	3.70	0.94	3.96	0.90
Education and Employment	Education	3.59	3.65	1.19	3.30	1.23	3.68	1.05	3.90	0.98
	Employment	3.05	3.34	1.16	2.63	1.09	3.01	0.93	3.23	1.00
	**Average overall score (30 indicators) **		**4.02**		**3.32**		**3.43**		**3.44**	

Relative Weights: ^1^Health importance - 37%; ^2^Potential for change - 27%; ^3^Gap characteristics - 26%; ^4^Public interest - 10%.

###  Agreement Between Participants’ Scores

 The degree of agreement across the 3 rounds is presented in Figure. Overall agreement on the 30 indicators rated in the first round was excellent (0.937); overall agreement on the 15 indicators rated in the second round and the 20 indicators rated in the third round was moderate (0.637 and 0.610, respectively). Agreement between 55 participants who rated the top 10 indicators during the third Delphi round was 0.751, representing a good agreement.

 Table S2 of Supplementary file 1 presents the difference of the average of all raters’ scores from the mean score given to each of the 30 indicators. Differences are very small, thus supporting the ICC estimate of good level of agreement.

## Discussion

 This study demonstrated how a diverse range of experts reached a consensus on the 10 most important national equity indicators. Clinical domains were represented by health risk factors (eg, cigarette smoking), and health outcomes (eg, infant mortality). System indicators were represented by healthcare resources (care accessibility and affordability). Three of the ten indicators were related to children, emphasizing the importance of early intervention to increase the likelihood of better health across the lifecourse.^49^ Several of the indicators, such as childhood injury or obesity, require collaboration between the healthcare and other sectors to improve equity. In line with the current equity approach that adopts an intersectional perspective,^50^ the selected indicators reflect inequity in multiple SDH – gender, ethnicity, religiosity, socio-economic status, education, income and geographic periphery – which often intersect, amplifying the burden of individual inequities.^3^ The fact that 40% of the selected indicators are related to system factors including access, affordability and resource distribution, points to a deep understanding of the contribution of healthcare services as well as socioeconomic conditions, both reflecting the layers of the SDH framework.^51^ During design of the process, the researchers hoped for reasonable compliance and envisioned an indicator set that would contribute to the inter-sectoral nature of health equity. The selection of indicators fulfilled and even went beyond those expectations.

 The level of agreement between participants was good in the first and second rounds and moderate in the final round. When analysing agreement on the 10 selected indicators among the 55 participants who completed the final rating, good agreement (ICC = 0.751) was achieved.^30^ Given the range of participants’ backgrounds, from different sectors, levels, regions, and ethnic groups, this level of consensus is encouraging for the future implementation of these indicators.^52^

 A Delphi process conducted to select indicators for evaluation of population health in Europe used a similar process,^44^ consulting with a wide range of experts to reach consensus on population health indicators, across economic and social factors, lifestyle and health behaviours, healthcare services and health outcomes among other fields. Infant mortality was also chosen as an indicator, as in the currently described process, with agreement between raters (Scott’s Pi inter-rater reliability coefficient) of 0.59. Overall agreement through the entire rating process ranged from 0.32-0.62.^44^ Another study used Delphi technique to reach a consensus on public health research priorities to address health inequities. Participants were receptive to the method and motivated to respond and the technique was found to be practical and effective in obtaining opinions from a wide range of experts. The most important research priorities were mental health, a healthy environment and health behaviours, two of which coincide with indicators chosen in the current study.^53^

 This process should be evaluated in light of anticipated possible barriers that could interfere with its successful completion: (1) Insufficient data to create an appropriate set of potential inequity indicators: some of the indicators were “created” de novo, by combining diverse data sources, as described in the Methods section, thus broadening the existing set of indicators that could be presented for rating. (2) Complexity of content that might deter participants without a background in health: A prerequisite to effectively achieving a consensus is that all participants have an equal opportunity to understand the issue under consideration and participate in a meaningful and fair way.^25^ The fairness of the process, whereby every participant had an equal voice, contributed to high engagement and compliance throughout the process. This is especially important in light of the heterogeneity of the participants, allowing a variety of viewpoints on the issue of health inequity,^54^ which is caused by multiple factors and requires multidisciplinary solutions. Indicators chosen through consensus of a broad range of people from different fields are more likely to be acceptable and ultimately implemented.^52^

 An online Delphi technique is especially challenging, as there is no opportunity for further explanations or dialogue, which could have been provided in a face-to-face Delphi format or other methodologies, bringing together experts in a workshop format.^55^ The presentation of the indicators in a clear and precise manner and request to rate the indicators in an easy-to-answer, closed format facilitated participation.^56^ The spontaneous feedback from participants and the high response rate indicated that the survey tool was suitable for a diverse audience.

 Enhancing the response rate is frequently addressed when conducting a Delphi survey.^57^ The very high response rate in the first two rounds and especially the high response in the last round, which took place during the first peak of the COVID-19 pandemic, may be seen as an indication of high commitment of the participants towards the consensus process. This may be attributed to several factors including personal engagement of the researchers with the participants in the initial contact and the reminders sent; to the fact that the process was commissioned by the MoH, thus enhancing the perception that the study results were more likely to be implemented and thus reduce inequities; to the invitation of participants based on their expertise in the topics under consideration, which may have contributed to participants’ perception of the process as meaningful and their contribution as relevant^58^; and to the accessibility and flexibility that the online consultation allowed.

 Analysis of the scores allocated to each of the four screening criteria indicates that participants allocated the lowest scores to the “potential for change” criterion, reflecting their awareness of the challenges of reducing health inequity, such as the need to incorporate social or policy components to interventions directed at individuals.^59^ An example of the challenging journey to improve health equity is Michael Marmot’s statement, ten years after the landmark review on health inequalities in England, that “the situation has become worse.”^60^ Moreover, the dynamics of the ratings between the rounds show that participants re-considered their own scoring in light of the feedback provided about the group’s rating. Most indicators were rated lower in successive rounds, compared with the first round. This might indicate that participants took the rating seriously as it approached the final round, and were aware of the difficulties implicit in reducing inequity in the domains and indicators presented in the process. However, participants did not make a significant change between rounds, with 8 of the 10 selected indicators being rated among the 10 leading indicators in the first round. The similarity between the rating rounds is compatible with the findings of another Delphi process.^44^

 The multi-level, multi-sectoral background of the participants brought diverse voices and perspectives to the consensus process, increasing the chance of selecting indicators that are acceptable to a wide audience. Effective intervention to increase health equity should be multi-sectoral, given the nature of health determinants.^61^ Involvement of stakeholders in the process, such as those responsible for reducing health equities in the four HMOs, might increase the chances of implementing this set of indicators to guide interventions aimed at reducing inequity.^52^

 Moreover, the active involvement of the regulating body – the MoH – in the research, facilitated collaborations during the preparation phase and also increased the likelihood of translation of the study outputs into measurable inequity reduction targets for the national healthcare system.

 This study bears several limitations. A relatively long interval between the second and third rounds, due to the burden imposed on participants during the peak of the COVID-19 outbreak, could have drastically reduced response rate, but in fact response rate remained high. The impact of such a health crisis on participants’ perspectives on the issues under consideration is difficult to determine. Despite extensive review of data sources, the 30 inequity indicators presented for selection do not cover all inequities in the health system. Moreover, data on health inequities from national registries are usually not up to date, and are often available with two or more years of lag, while other sources may be more up to date. All the data presented in the research were the latest available. Only a few indicators suggested by participants were suitable for inclusion in the selection process and based on reliable data, although none were highly rated. Given the number and diversity of the 30 indicators, we believe that the process covered most of the indicators that were available and suitable to enter the process. While efforts were made to recruit a diverse panel of experts, including those from minority groups, and from the periphery, the Delphi process by nature engages with experts in various fields, and may therefore exclude those with a language barrier, low literacy, or lack of access to technology. We recommend the design of a platform that allows disadvantaged groups to participate and bring the voices of those who need it most. Each equity indicator was analyzed and presented as relative difference. While the use of “absolute difference” (or absolute inequality) could add important information, we wanted our study to reflect the widely used measurements of inequities in Israel to further enhance participation and the chances of implementation of the agreed upon indicators. Once the indicators are well-established, they can be additionally presented in absolute terms. Last, but not least, SDH were somewhat underrepresented among the proposed indicators and none were included in the 10 most highly rated equity indicators. The impact of SDH on health has become even more relevant during the COVID-19 pandemic, which most significantly affected people with poor housing and working conditions, lower health literacy and pre-existing co-morbidities that exposed them to higher risks.^62,63^ The pandemic, which has exacerbated disparities based on SDH, may act as a catalyst to give SDH a more prominent place within the health system.

 A challenging and critical stage in transforming the set of indicators from “a grocery list” to a compass pointing in the “right direction” is the implementation stage. The MoH – who initiated the process – is accountable for adopting the indicator set and using it to guide governmental decision-making and inter-ministerial strategies. More specifically, the MoH has committed to translate the general definition of each of the 10 equity indicators into detailed operative definitions and to design dedicated computerized infrastructures to present these indicators on the MoH public site. Healthcare providers will be encouraged to use the indicators to guide their policy while the MoH will monitor national and regional progress towards reduction in inequities. Budgets will be allocated to help reduce regional inequities in healthcare infrastructures and health inequities between the least and the most deprived regions. Governmental policies, standards and regulations will reflect these strategies; ongoing monitoring and public reporting of these measures should be coupled with the setting of targets for the reduction of the highlighted inequalities.

## Conclusion

 The Delphi technique provided an easy to use, fair, and relatively quick method for consulting with a variety of experts and public representatives to choose a set of national equity indicators.

 Policy-makers in countries which have not yet set a unified indicator set or those who wish to renew or expand an existing set can utilize the framework described here to build their own process, taking advantage of its strengths including evidence-based data, participatory nature, and low investment of time and resources.

 The diverse set of indicators selected in this research could promote inter-sectoral collaboration both at the level of ministries and in the field. We hope that the development of a national set of equity indicators will produce a ripple effect, creating additional processes that will gradually expand public and professional involvement in the quest towards a more equitable healthcare system.

## Ethical issues

 The study protocol was approved by the Institutional Ethics Committee (Approval number 10370984).

## Competing interests

 Authors declare that they have no competing interests.

## Authors’ contributions

 All authors interpreted the data and edited and approved the final article. RWM, PFB, SA, LV, GP, MS, and OL conceptualized and designed the study, drafted the initial manuscript, and reviewed and revised the manuscript. AZ, VM, MS, and RWM designed the methods section, analyzed the data and reviewed and revised the manuscript. RWM and PFB critically reviewed the manuscript for important intellectual content.

## Funding

 The research was funded by a grant from The Israel National Institute for Health Policy Research (Grant number 0601445671).

## Availability of data and materials

 All data generated or analysed during this study are included in this published article.

## Supplementary files

Supplementary file 1 contains Tables S1 and S2.
