# Supplementary material for: Developing a National Set of Health Equity Indicators Using a Consensus Building Process
Source: Int J Health Policy Manag. 2021 Jun 23;11(8):1522–32. doi: 10.34172/ijhpm.2021.54 (PMC9808363; doi:10.34172/ijhpm.2021.54)
Supplement: Supplementary file 1 — contains Tables S1 and S2. [file ijhpm-11-1522-s001.pdf]

Article title: Developing a National Set of Health Equity Indicators Using a Consensus Building Process

Journal name: International Journal of Health Policy and Management (IJHPM)

Authors' information: Rachel Wilf-Miron[1,2], Shlomit Avni[3], Liora Valinsky[4], Vicki Myers[1], Arnona Ziv[1], Gidi Peretz[3], Osnat Luxenburg[5], Mor Saban[1]*, Paula Feder-Bubis[6]

[1]The Gertner Institute for Epidemiology and Health Policy Research, Sheba Medical Center, Ramat Gan, Israel.

[2]School of Public Health, Sackler Faculty of Medicine, Tel Aviv University, Tel Aviv, Israel.

[3]Strategic and Economic Planning Administration, Ministry of Health, Jerusalem, Israel.

[4]Public Health Services, Ministry of Health, Jerusalem, Israel.

[5]Medical Technology, Health Information and Research Directorate, Ministry of Health, Jerusalem, Israel.

[6]Department of Health Policy and Management, Faculty of Health Sciences & Guildford Glazer Faculty of Business and management, Ben Gurion University of the Negev, Beersheba, Israel.

(*Corresponding author: morsab1608@gmail.com)

Supplementary file 1

Table S1. Relative differences by selected social determinants of health, for the 30 indicators presented for rating (10 final indicators highlighted)

Domain	Indicator / SDH*	Age	Gender	Geographic periphery	SES/Income	Education	Ethnicity	Ethnicity and socioeconomic status	Religiosity
Health promotion and disease prevention	**Smoking ≥ 21 years old**				RR**=1.4 (lower to higher SES*** quartile)	RR=1.36 (Lower education to higher education)	RR=1.81 (Male Arabs to Male Jews)		
	**Mammography screening**				RR=1.13 (higher to lower SES quartile)		RR=1.36 (Female Arabs to female Jews)		RR=1.93 (Secular to ultra-Orthodox)
	**Colorectal cancer screening**	RR=1.32 (Older population (70-74) younger population (50-54))			RR=1.19 (higher to lower SES quartile)				

	**Achieving physical activity target**		RR=1.13 (Male to female)		RR=1.51 (High income to low income)	RR=1.51 (Higher education to lower education)	RR=1.53 (Jews to Arabs)<br><br>RR=1.38 (Male Jews to Male Arabs)<br>RR=1.87 (Female Jews to female Arabs)		RR=1.35 (Secular to ultra-Orthodox)
	**Antibiotic prophylaxis - hip fracture surgery**			RR=1.17 (Center to periphery)					
	**MMRV routine vaccinations**			In Arabs- RR=1.19 (Center to periphery)			RR=1.34 (Arabs to Jews)	In Jews- RR=1.11 (higher to lower SES quartile)<br><br>In Arabs- RR=1.13 (higher to lower SES quartile)	
	**DTaP-IPV-HIB routine vaccinations**			In Jews- RR=1.17 (Center to periphery)			RR=1.23 (Arabs to Jews)	In Jews- RR=1.55 (higher to lower SES quartile)<br><br>In Arabs- RR=1.1 (higher to lower SES quartile)	
	**First well baby care visits in Maternal and Child Clinics**						RR=1.47 (Arabs to Jews)	In Jews- RR=4.07 (higher to lower SES quartile)<br><br>In Arabs- RR=1.66 (higher	

								to lower SES quartile)	
	**Post-partum depression**				RR=2.25 (unemployed female to employed female)	RR=2.45 (Lower education to Higher education)	RR=2.96 (Female Arabs to Female Jews)		
	**Poor perceived health status**		RR=1.20 (Female to Male)			RR=3.64 (Lower education to higher education)	RR=1.57 (Arabs to Jews)		RR=1.52 (Ultra- Orthodox to Secular)
	**Poor dental health - children**								
	**Poor dental health - adults**				RR=1.99 (lower to higher SES quartile)<br><br>RR=1.59 (unemployed females to employed females)	RR=2.51 (Lower education to Higher education)	RR=1.72 (Jews to Arabs)		
Acute and chronic morbidity	**Poor diabetes control** (%HbA1c>9)			RR=1.17 (Peripheral vs central residence)	RR=2.5 (lower to higher SES quartile)		RR=2.5 (Arabs to Jews)		
	**Obesity in 7th grade students** (BMI>97%)			Southern (3.11(RR= vs central region)	RR=1.26 (lower to higher SES quartile)		RR=1.57 (Arabs to Jews)		
	**Obesity in adults** (BMI≥30)		RR=1.12 (Female to Male)	1.38 (RR= Southern vs central region)			RR=1.76		

							(Female Arabs to Female Jews)<br><br>RR=1.20 (Male Arabs to Male Jews)		
	**End stage renal disease patients on dialysis**				RR=2.1 (lower to higher SES decile)				
	**Stroke and Incidence care**				RR=2.64 (lower to higher SES decile)		RR=2.5 (Arabs to Jews)		
	**Severe disability (adults)**				RR=2.63 (Low income to High income)	RR=2.83 (Lower education to higher education)	RR=1.96 (Arabs to Jews)<br><br>RR=1.69 (Female Arabs to Jewish female)		
	**Lung cancer – stage at diagnosis**				In male- RR=1.47 (lower to higher SES)			In male Jews- RR=1.59 (higher to lower SES quartile)<br><br>In female Jews- RR=1.15 (higher to lower SES quartile)	
	**Colorectal cancer – stage at diagnosis**	RR=1.08 (Older population (70-74)	RR=1.11 (Male to Female)						RR=1.02 (Ultra- Orthodox to Secular)

		younger population (50-54))							
	**Breast cancer -stage at diagnosis**	RR=1.09 (Older population (70-74) younger population (50-54))			RR=1.12 (higher to lower SES		RR=1.15 (Jews to Arabs)		RR=1.07 (Secular to Ultra- Orthodox)
Life expectancy and mortality	**Fatal unintentional injury** among 0-17 year olds			RR=3.11 Southern vs Tel Aviv region	RR=2.48 (lower to higher SES quartile)		RR=1.79 (Arabs to Jews)		
	**Infant mortality**			RR=2.57 (Southern (vs central region)			RR=1.57 (Arabs to Jews)		
	**Life expectancy**		RR=1.11 (Female to Male)	2.8 years (Judea and Samaria (District vs south		Male- 4.4 years (Lower education to higher education)<br><br>Female- 4.7 years (Lower education to higher education)	3.7 years (Male Jews to Male Arabs)<br><br>3 years (Female Jews to Female Arabs)		
	**Suicide**	In male- RR=3.84 (Older population (75+) younger population (15-24)	RR=5.05 (Male to Female)						

		In female- RR=2.79 (Older population (75+) younger population (15-24)							
Accessibility and affordability of healthcare	**Waiting time for psychotherapy**			166 days longer in Haifa District vs South					
	**Inability to afford treatment or prescribed medication**			RR=5.5 (Low income to High income)		RR=3.23 (Lower education to higher education)	RR=3.9 (Arabs to Jews)		
	**Distribution of healthcare personnel**			Physician-<br><br>2.45 (RR= Central region vs North)<br><br>Nurse-<br>1.97 (RR= Central region vs North)					
	**Distribution of Hospital beds**			General beds- RR=1.49 Jerusalem vs South region<br><br>ICU beds- RR=1.71 Haifa vs north region<br><br>Rehabilitation beds- RR=4.75 Tel Aviv vs North region					

Education and Employment	**Education - PISA scores among 15-year-olds**			Higher to lower SE quartile RR=1.78			Jews/Arabs: Reading literacy RR=1.4; Science literacy 1.31		
	**Employment rate**						RR=1.06 (Male Jews to Jewish female)<br><br>RR=1.16 (Male Jews to Male Arabs)<br><br>RR=2.32 (Jews female to Arab female)		In males- RR=1.76 (Secular to Ultra- Orthodox)<br><br>In females- RR=1.09 (Secular to Ultra- Orthodox)

*SDH=social determinants of health

**RR=rate ratio

***SES=Socioeconomic status

Table S2. Mean raters' differences from mean score, by indicator

	Absolute	Positive*	Negative**
**Indicator**			
Cigarette smoking	0.101	0.092	-0.101
Achieving physical activity target	0.081	0.051	-0.081
Screening for breast cancer	0.141	0.072	-0.141
Screening for colorectal cancer	0.090	0.059	-0.090
Antibiotic prophylaxis for hip fracture surgery	0.112	0.081	-0.112
Routine child immunization	0.103	0.059	-0.103
First visit at well-baby clinic	0.073	0.064	-0.073
Post-natal depression	0.081	0.074	-0.081
Perceived health status	0.089	0.089	-0.083
Dental hygiene, children	0.130	0.079	-0.130
Dental hygiene, adults	0.106	0.068	0.106
Adult obesity	0.123	0.097	-0.123
Severe physical disability, adults 20+	0.093	0.063	-0.093
Diabetes care	0.132	0.087	0.132
Prevalence of obesity, children	0.084	0.040	-0.084
Incidence and treatment of stroke	0.078	0.078	-0.069
Prevalence of end stage renal disease, requiring dialysis	0.093	0.079	-0.093
Stage of diagnosis, lung cancer	0.084	0.084	-0.077
Stage of diagnosis, colon cancer	0.077	0.074	-0.077
Stage of diagnosis, breast cancer	0.061	0.057	-0.061
Infant mortality	0.093	0.057	-0.093
Life expectancy	0.089	0.066	-0.089
Fatal childhood injury	0.070	0.070	-0.061
Suicide	0.082	0.045	-0.082
Access to psychotherapy	0.082	0.082	-0.070
Ability to afford care	0.073	0.043	-0.073
Distribution of healthcare personnel	0.132	0.062	-0.132
Distribution of hospital beds	0.096	0.061	-0.096
Education	0.073	0.056	-0.073
Employment	0.070	0.062	-0.070

*Positive - Mean positive difference of ratings from the mean score

**Negative - Mean negative difference of ratings from the mean score
